# Trends and predictors of changes in renal function after radical nephrectomy for renal tumours

**DOI:** 10.1186/s12882-024-03601-2

**Published:** 2024-05-21

**Authors:** Yongchao Yan, Yunbo Liu, Bin Li, Shang Xu, Haotian Du, Xinning Wang, Yanjiang Li

**Affiliations:** The Affiliated Hospital of Qingdao University, Qingdao, China

**Keywords:** Renal function, Short-term changes, Long-term prognosis, AKI, CKD

## Abstract

**Background:**

Chronic kidney disease (CKD) is a common postoperative complication in patients who undergo radical nephrectomy for renal tumours. However, the factors influencing long-term renal function require further investigation.

**Objective:**

This study was designed to investigate the trends in renal function changes and risk factors for renal function deterioration in renal tumour patients after radical nephrectomy.

**Methods:**

We monitored changes in renal function before and after surgery for 3 years. The progression of renal function was determined by the progression and degradation of CKD stages. Univariate and multivariate logistic regression analyses were used to analyse the causes of renal function progression.

**Results:**

We analysed the data of 329 patients with renal tumours who underwent radical nephrectomies between January 2013 and December 2018. In this study, 43.7% of patients had postoperative acute kidney injury (AKI), and 48.3% had CKD at advanced stages. Further research revealed that patients’ renal function stabilized 3 months after surgery. Additionally, renal function changes during these 3 months have a substantial impact on the progression of long-term renal function changes in patients.

**Conclusion:**

AKI may be an indicator of short-term postoperative changes in renal function. Renal function tests should be performed in patients with AKI after radical nephrectomy to monitor the progression of functional impairment, particularly within the first 3 months after radical nephrectomy.

## Key learning points

1. After unilateral nephrectomy, 48.3% of the patients had an increase in CKD grade.

2. Changes in short-term renal function have an important impact on long-term renal function.

3. We should monitor and intervene in the short-term renal function of patients who undergo unilateral nephrectomy to prevent poor long-term renal function.

## Introduction

Currently, radical nephrectomy is one of the options for treating malignant renal tumours [1]. Removal of the kidney inevitably reduces the functional renal parenchyma, resulting in loss of renal function [2]. Previous studies have demonstrated that older age, high comorbidity rates, and low preoperative estimated glomerular filtration rate (eGFR) are associated with chronic kidney disease (CKD) after radical nephrectomy [3, 4].

Postoperative CKD in patients undergoing radical nephrectomy is associated with an increased risk of all-cause and cardiovascular disease-related mortality [5]. Previous studies have demonstrated that CKD results in increased proteinuria and blood pressure, which are associated with an increased risk of cardiovascular disease and all-cause mortality in the general population [6]. Observing and recording changes in patients’ eGFR after radical nephrectomy is crucial, and early measures should be taken to prevent CKD. Moreover, these findings can help improve our understanding of the changes in renal function in patients after surgery and explore the indicators related to the progression of renal function impairment.

Therefore, the present study aimed to examine the trends in renal function changes and identify risk factors for renal function deterioration in patients with renal tumours after radical nephrectomy. Furthermore, key nodes involved in renal function changes were also identified.

## Materials and methods

### Patient selection

As this publication is a report that contains no identifiable content to the patient, this publication was exempt from ethical approval by the Human Research Protection Program (HRPP) and its Institutional Review Board (IRB) at the Ethics Committee of the affiliated hospital of Qingdao University. The requirement for written informed consent was waived by the Institutional Review Board (IRB) of The Affiliated Hospital of Qingdao University owing to the retrospective nature of the study. Using the scientific research big data platform of our hospital, we screened patients who underwent unilateral nephrectomy for renal tumours between 2013 and 2018. To ensure the completeness of the data, 329 patients were screened according to their postoperative follow-up compliance.

### Patient data and outcome measurements

The demographic and baseline characteristics of the patients related to renal function and procedure-related data were obtained from the electronic medical records maintained at the hospital (Table 1); preoperative serum creatinine values and those at postoperative week, as well as at 3, 6, 12, and 36 months after surgery, were collected. Additionally, eGFR was calculated using the Modification in Diet and Renal Disease (MDRD) formula [7]. We defined postoperative AKI as recommended by the Kidney Disease Improving Global Outcomes 2012 guidelines: an increase in plasma creatinine > 26.5 µmol/L within 48 h or an increase in plasma creatinine > 1.5 fold the baseline, which is known or presumed to have occurred within the prior 48 h postoperatively [8]. Renal function was staged as CKD I–V according to the American Standard Stages of Renal Function [7].

**Table 1:** Demographic and baseline characteristics of the patients

Variables	Patients
Age, year	58(51–65)
Male, n	211(64.2)
Hypertension, n	132(40.1)
Diabetes mellitus, n	43(13.0)
Surgical methods	
Open,n	59(17.9)
Laparoscopy,n	270(82.1)
Preoperative eGFR, mL/min/1.73 m2	
eGFR ≥ 90 mL/min/1.73m2	208(63.2)
eGFR <90 mL/min/1.73m2	121(36.8)
eGFR <60 mL/min/1.73m2	10(3.0)
BMI,Kg/m^2^	25.22 ± 5.49
Tumor size, cm	5.98 ± 2.68
Clinical stage, n	
T1a	129(39.2)
T1b	128(38.9)
T2a	50(15.1)
T2b	22(6.6)
the ratio of postoperative creatinine to preoperative creatinine	1.33 ± 0.36
Hypotensive shock,n	6(1.8)
AKI,n	144(43.7)
Stage of AKI	
1,n	128(38.9)
2,n	15(4.5)
3,n	1(0.3)
Average eGFR decline at 36 months,mL/min/1.73 m2	23.76
Upgrading CKD staging,36 months,n	159(48.3)

AKI = acute kidney injury; eGFR = estimated glomerular filtration rate; BMI = Body Mass Index; AKI stages are divided according to KDIGO 2012

### Statistical methods

SPSS software (version 25.0; IBM Corp., Armonk, NY, USA) was used to analyse the data, and a line chart was used to demonstrate the trends in the data. Normally distributed data are presented as the mean ± standard deviation, and non-normally distributed data are presented as the median (25th, 75th percentile). For all analyses, *p* < 0.05 was considered indicative of statisticaly significance.

### Study design

**Fig. 1:**
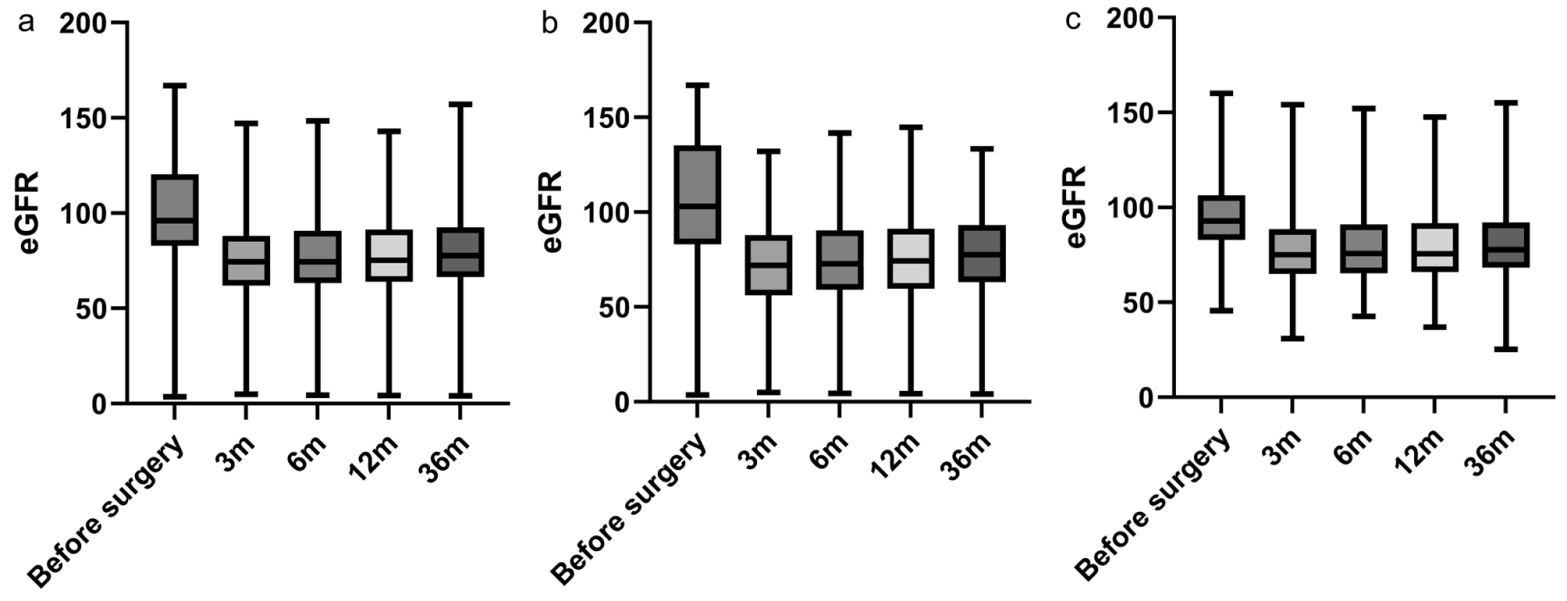
The box plots of eGFR at different time points. m = month **a**: eGFR of all patients at different time periods. **b**: eGFR of patients who don’t experienced AKI at different time periods **c**: eGFR of patients who experienced AKI at different time periods

**Fig. 2:**
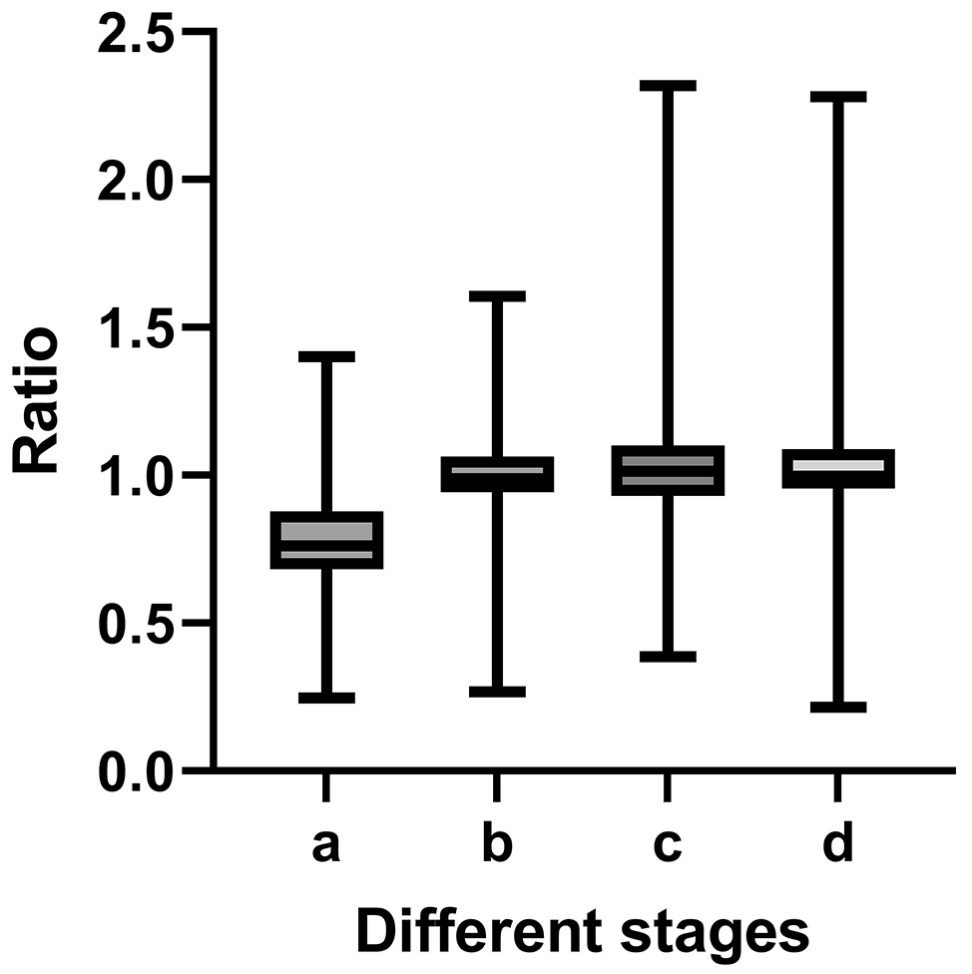
The rate of change at different stages. **a**: The ratio of the 3-month eGFR to the preoperative eGFR. **b**: The ratio of the 6-month eGFR to the 3-month eGFR. **c**: The ratio of the 12-month eGFR to 6-month eGFR. d: The ratio of the 36-month eGFR to the 12-month eGFR

First, the MDRD formula was used to calculate changes in renal function at 3, 6, 12, and 36 months after surgery. We plotted the data of the 329 patients as box plots of eGFR at different time points (Fig. 1). The rate of change at different stages was plotted in a box plot (Fig. 2). We categorized the patients into groups of 0–3, 3–6, 6–12, and 12–36 months. We utilized box plots to observe the changes in renal function among patients, providing insights into the trends of these changes.

Second, we assessed the patients’ CKD stages and determined whether the stage had increased after 36 months as a measure of renal function progression. Trend plots showed that the changes in postoperative renal function were most obvious in the early postoperative period. Subsequently, we compared the eGFR value in the early postoperative period with the preoperative value as the rate of change in renal function. Moreover, we examined the relationships between the early rate of change in renal function, age, sex, tumour size, hypertension status, surgical methods, hypotensive shock, AKI, diabetes mellitus status, and CKD development during the 36 months after surgery using multivariate Cox proportional hazard regression analysis as displayed in Table 2.

**Table 2:** Logistic regression models of influencing factors of long-term renal function

	Univariate analysis				Multivariate analysis		
Variables	OR	95% CI	*P*-values		OR	95% CI	*P*-values
Preoperative Renal function	1.006	1.000−1.013	0.066		1.007	0.999–1.015	0.088
Hypertension	0.808	0.520–1.257	0.344		0.846	0.492–1.457	0.547
Diabetes	1.351	0.706–2.584	0.364		1.807	0.826–3.954	0.138
Tumor size	0.925	0.858–0.998	0.046		0.917	0.833–1.010	0.078
Gender	2.015	1.270–3.195	0.003		1.771	1.048–2.992	0.033
BMI	1.012	0.972–1.054	0.562		0.998	0.954–1.045	0.938
Age	1.033	1.012–1.055	0.002		1.019	0.994–1.046	0.140
Rate of change in renal function at three months	0.007	0.001–0.033	< 0.0001		0.002	0.001–0.0015	< 0.0001
Surgical methods	0.812	0.461–1.430	0.470		1.390	0.664–2.913	0.383
Hypotensive shock	0.716	0.260–1.970	0.517		0.733	0.199–2.690	0.639
Different stages of AKI	1.574	1.088–2.278	0.016		0.755	0.471–1.210	0.242

Third, we discovered that the early rate of change in renal function was an important factor affecting long-term renal function. Therefore, we further explored the factors influencing the rate of early renal function changes as displayed in Table 3. Linear regression was used to analyse the relationships between preoperative renal function, age, tumour size, BMI, the ratio of postoperative creatinine to preoperative creatinine, and the rate of early postoperative renal function change.

**Table 3:** Linear regression analysis of Influencing factors of short-term renal function change rate

	Univariate analysis			Multivariate analysis	
Variables	R	*P*-values		R	*P*-values
Preoperative Renal function	-0.403	< 0.0001		−0.189	0.0004
Tumor size	0.066	0.234		−0.029	0.522
Age	−0.204	< 0.0001		−0.164	0.0004
Ratio of postoperative creatinine to preoperative creatinine	-0.555	< 0.0001		−0.439	< 0.0001

## Results

The demographic and perioperative parameters are displayed in Table 1. The incidence of CKD escalation was 48.3% 36 months postoperatively. The incidence of postoperative AKI escalation was 43.7%.

Based on the eGFR of the postoperative patients, we plotted the lines in Figs. 1 and 2. The renal function of most patients decreased rapidly within 3 months after surgery and then stabilized.

Table 2 shows that the main factors influencing long-term renal function at this stage were the rate of change in renal function (*p* < < 0.0001, odds ratio (OR) = 0.002) and sex (*p* = 0.033, OR = 1.771). The greater the decline in renal function, the poorer the long-term renal function prognosis. However, we found that AKI grading, surgical approach, and intraoperative hypotension do not affect long-term renal function.

Additionally, Table 3 shows that preoperative renal function (*p* = 0.004, *r* = 0.131), age (*p* = 0.006, *r*=-0.128), and the ratio of postoperative creatinine to preoperative creatinine (*p* < 0.0001, *r*=-0.528) were correlated with the rate of change in renal function within 3 months after surgery. However, preoperative creatinine and age were weakly correlated. A strong correlation was observed between the ratio of postoperative creatinine to preoperative creatinine and the severity of AKI; a high ratio indicated a severe degree of AKI. Moreover, the greater this ratio, the more substantial the decline in renal function.

## Discussion

In a retrospective cohort with 36 months of follow-up, we calculated patient eGFR from the collected data and plotted the trend of postoperative renal function. Moreover, we used multiple-factor regression analysis to evaluate the factors influencing the long-term progression of renal function. We also plotted curves depicting renal function changes in different patients and identified key nodes in their trajectories.

The poor progression of the patient’s long-term renal function after surgery was revealed, and the CKD grade increased. Studies have demonstrated that CKD after radical nephrectomy is associated with all-cause mortality and the incidence of cardiovascular disease [5]. Furthermore, after radical nephrectomy, patients choose different adjuvant therapies, such as chemotherapy, immunotherapy, and radiotherapy, according to the stage and grade of the tumour [9]. These treatments have specific requirements for renal function. Therefore, determining the factors influencing long-term renal function after surgery is crucial and necessitates the implementation of corresponding intervention measures in the early stages to prevent the occurrence of CKD.

Gender is an important factor influencing the occurrence of CKD, as shown by our data. Compared to women, men have a greater rate of all-cause and cardiovascular mortality, an increased risk of CKD progression, and a steeper decrease in the eGFR [7]. The reasons for potential sex-related differences in CKD progression are unclear, but differences in potential risk factors and sex hormone effects have been proposed [10]; for example, animal studies have shown a protective, anti-inflammatory effect of oestrogen on podocytes and a reduced permeability effect on the glomerular endothelium [11, 12].

Research has proven that radical nephrectomy gradually restores renal function in patients [2, 13]. Figures 1 and 2 show that the renal function of most patients decreased after surgery. Our research also revealed that in most patients, postoperative renal function gradually declined within 3 months of surgery and remained stable after 3 months. Subsequently, the renal function of most patients gradually stabilized 3 months after surgery. For most patients, the long-term trajectory of renal function can be reasonably predicted based on the renal function observed at the 3-month mark after surgery. Additionally, one study demonstrated that 49% of patients recovered their preoperative eGFR within 2 years of radical nephrectomy for renal cell carcinoma [14]. Therefore, we believe that 3 months after surgery is the period during which renal function is most likely to be impacted, and the protection of renal function at this stage is crucial.

In addition, Table 3 shows that the most important factor for long-term renal function was the rate of change in the eGFR during the early postoperative period. During this period, owing to the removal of the affected kidney, the contralateral healthy kidney compensates with hyperplasia, thus supplying the entire body [15]. Therefore, the kidney remaining during this period is extremely fragile owing to the workload. According to the Remnant Kidney Model, after the reduction of kidney mass, the workload for the remaining nephrons results in progressive renal damage leading to terminal renal failure [16]. Therefore, healthy kidneys may undergo permanent changes during this time, with important implications for long-term kidney function. This is also reflected in the data obtained in our study. During this period, the influence of the rate of change in the eGFR on long-term renal function surpassed that of age, and it subsequently stabilized. Hence, this period represents the optimal time for predicting and safeguarding renal function.

Several studies have demonstrated that postoperative AKI is a common complication after radical nephrectomy [17, 18]. According to a literature review, 41–47% of patients develop clinical AKI within 48 h after radical nephrectomy [19], consistent with the incidence of AKI reported in our cohort. Some studies have suggested that postoperative AKI after radical nephrectomy is associated with the deterioration of long-term renal function [20, 21].

In our research, we studied the relationship between the ratio of postoperative creatinine to preoperative creatinine and the rate of change in renal function 3 months after surgery. The greater the ratio, the more severe the degree of AKI [8]. Therefore, AKI can be used as an indicator of short-term postoperative renal function, and the compensatory capacity of the kidney can be used to further predict long-term renal function.

However, we believe that postoperative AKI after radical resection differs from postoperative AKI in other typical clinical situations. In other typical clinical cases, postoperative AKI refers to the destruction of the renal parenchyma and reduction of glomeruli caused by ischaemia, inflammation, and immune factors [22, 23]. Hypotensive shock-induced AKI mainly occurs in clinical situations such as cardiovascular surgery and kidney transplantation. The pathogenesis is primarily ischemia-reperfusion injury, leading to renal hypoxia-ischemia necrosis and long-term fibrosis [24]. he causes of AKI are numerous, among which, renal ischemia-reperfusion injury caused by perioperative hypotension is a significant factor leading to AKI and can result in long-term renal function decline. However, data from this study indicate that perioperative hypotension is not a risk factor for CKD, which may be related to the lower incidence of hypotensive shock in our data. The development of postoperative AKI after radical nephrectomy does not damage the contralateral healthy kidney and prompts compensatory hyperplasia in the remaining kidney [25]. Immediately after nephrectomy, a 40% increase in blood flow and the GFR was observed in the kidneys [26]. This progresses to affect the glomeruli, increasing the high-pressure experienced by the nephron filters and resulting in compensatory enlargement of the glomeruli [25].

Once the glomerular volume reaches a certain threshold, glomerulosclerosis, hypertension, proteinuria, and renal failure may occur [27]. An increase in renal blood flow and the GFR leads to increased oxygen consumption, which in turn leads to tissue hypoxia. These conditions induce hypoxia-inducible Factor 1α and vascular endothelial growth factor. Hypoxia also induces small tube phosphatases and tension homologues, leading to tubular regeneration and repair [28]. Therefore, serum creatinine levels rapidly increase after unilateral nephrectomy, leading to AKI. To confirm that postoperative creatinine may be attributed exclusively to nephrectomy without damage to the contralateral kidney, AKI markers should be introduced into clinical practice to quantify any suffering of the contralateral kidney [19, 29].

The occurrence and severity of AKI after nephrectomy can be used as detection indicators, enabling more informed assessments and the formulation of programs aimed at enhancing short-term renal function recovery. These strategies include reducing the prerenal load, controlling blood pressure, and eating properly. Moreover, they contribute to long-term renal function. The recent literature revealed remaining kidney parenchymal (contralateral kidney) could predict the new baseline eGFR. It could be a tool for decision choice of treatment for the RCC patients [30]. Further studies are needed.

However, this study has the following limitations that need to be addressed in future research. First, it should further expand the sample size for follow-up and include novel biomarkers of AKI, cystatin C and/or NephroCheck test [19, 29]. Secondly, due to the inconvenience of clinical sample collection, urinary volume is not included in the diagnostic criteria for AKI.

Conclusion.

In conclusion, postoperative renal function in most patients gradually declined within 3 months of surgery and remained stable after 3 months. More attention should be given to AKI patients during the first 3 postoperative months, especially for those with decreased renal function. Additionally, the implementation of appropriate measures is very important for the recovery of patients with long-term renal function impairment. These measures can also help when deciding to adopt treatment options that may be highly demanding in terms of renal function.

## Data Availability

The datasets used and/or analysed during the current study available from the corresponding author on reasonable request.

## Declarations

### Animal studies

N/A.

### Registry and the Registration No. Of the study/trial

N/A.
